# Experimental Investigations on the Flexural Responses of Reinforced Rubberized Concrete Beams with High Rubber Contents and Longitudinal Reinforcement Ratios

**DOI:** 10.3390/ma19183947

**Published:** 2026-09-17

**Authors:** Petru Mihai, Ana-Maria Toma, Septimiu-George Luca, Aliz-Eva Mathe, Vasile-Mircea Venghiac, Irina Lungu

**Affiliations:** 1 Faculty of Civil Engineering and Building Services, The “Gheorghe Asachi” Technical University of Iasi, No. 1, Prof. dr. doc. D. Mangeron Street, 700050 Iasi, Romania; petru.mihai@academic.tuiasi.ro (P.M.); septimiu-george.luca@academic.tuiasi.ro (S.-G.L.); vasile-mircea.venghiac@academic.tuiasi.ro (V.-M.V.); 2 Faculty of Civil Engineering, Technical University of Cluj-Napoca, No. 15th, Constantin Daicoviciu Street, 400020 Cluj-Napoca, Romania; aliz.mathe@mecon.utcluj.ro

**Keywords:** waste tyre rubber, rubberized concrete, reinforced concrete beams, flexural behaviour, longitudinal reinforcement ratio, ductility, crack pattern

## Abstract

**Highlights:**

- Rubber replacement up to 60% by volume enhances beam ductility and deflection capacity at 1.5% reinforcement.
- Compression zone crushing, not rubber content, governs failure once reinforcement ratio reaches 2.0%.
- Rubberized concrete beams meet Eurocode 2 strain limits despite a lower compressive strength class.

**Abstract:**

The accumulation of end-of-life tyres represents a growing environmental burden, motivating the incorporation of tyre rubber as a partial aggregate replacement in concrete. However, the flexural performance of reinforced rubberized concrete (RuC) beams at high rubber contents and elevated longitudinal reinforcement ratios remains insufficiently investigated. This study experimentally examined the flexural behaviour of RC beams cast with conventional concrete and RuC incorporating 40% and 60% replacement of small coarse aggregate by volume, at longitudinal reinforcement ratios of 1.5% and 2.0%, approaching the over-reinforced range. Eighteen beams were tested under four-point bending until failure by concrete crushing, with deflection, crack development, and material properties monitored throughout. Rubber incorporation reduced the modulus of elasticity and compressive strength, corresponding to a Eurocode 2 class downgrade from C45/55 to C35/40, while increasing peak axial strain. At 1.5% reinforcement, increasing rubber content progressively enhanced deflection capacity and ductility, with RuC60 beams reaching a ductility coefficient of 2.52 versus 2.43 for conventional beams. At 2.0% reinforcement, this benefit was suppressed, with ductility converging to 1.40–1.47 across all mixes as compression zone crushing increasingly governed failure. These findings indicate that the structural benefits of high-rubber-content RuC are most pronounced in moderately reinforced beams and diminish as sections approach the over-reinforced regime, informing the applicability envelope of RuC for load-bearing flexural elements.

## 1. Introduction

The accumulation of end-of-life tyres has become one of the most pressing environmental challenges of the present day. An estimated one billion waste tyres are generated worldwide each year, a figure projected to approach 1.2 billion by 2030 [1]. Conventional disposal routes, landfilling and incineration, are increasingly untenable: the former is constrained by the rapid depletion of available disposal capacity, while the latter contributes to air pollution. Furthermore, waste tyre rubber is non-biodegradable and possesses an exceptionally long service life, so that once discarded, it persists in the environment for decades [1,2]. Recovering rubber from these tyres and using it as a partial replacement for natural aggregates in concrete, producing so-called rubberised concrete (RuC), has therefore attracted sustained attention from the civil engineering community as a means of valorising this waste stream [1,2]. Given that approximately five billion tonnes of concrete are produced worldwide every year, the benefit of this substitution is twofold. Ecologically, it diverts a substantial volume of waste tyres from landfills, while reducing the demand for virgin aggregates, supporting the transition towards more sustainable construction materials [3,4]. From an engineering standpoint, the addition of rubber can enhance dynamic and durability-related properties, such as ductility and damping capacity [3], as well as resistance to chloride ion penetration and carbonation [2], together with improved toughness, impact resistance and thermal, electrical, and acoustic insulation [1]. This solution is nevertheless accompanied by technical limitations, among which the most significant is a decline in mechanical strength at high rubber contents [5].

Beyond its mechanical implications, the substitution of natural fine aggregate with tyre-derived rubber chips carries a modest cost penalty, driven mainly by the shredding, sizing and cleaning required to convert scrap tyres into usable aggregate. Quantitative cost comparisons between crumb rubber concrete and ordinary Portland cement concrete of equivalent design strength report an average production cost increase of approximately 6–7% for rubber replacement below 20% of the fine aggregate volume [6], attributable chiefly to the higher unit price of processed rubber relative to natural sand, rather than to the cement content, which remained the dominant cost driver overall. This penalty is expected to diminish as tyre-processing infrastructure matures and rubber recycling markets scale up.

The effects of rubber content on the mechanical properties of RuC have been extensively documented. Across a range of concrete types, both the compressive strength and the modulus of elasticity have been consistently reported to decrease with increasing rubber content, the loss in compressive strength following an approximately linear trend [2,7]. Parametric studies indicate that rubber can replace up to about 20% of the total aggregate volume before a pronounced strength loss occurs [2], a reduction primarily attributed to the weak interfacial transition zone (ITZ) between the rubber particles and the surrounding cementitious matrix [1]. At the microstructural level, the rubber acts as an inert filler that dilutes the formation of C–S–H gel and inhibits cement hydration, increasing porosity, particularly the macropore fraction, and weakening the continuity of the microstructure. At the macroscopic level, the low stiffness of the rubber particles induces stress concentrations, and their hydrophobic surface promotes cracking along the ITZ. Together with the increased air content, these microstructural and interfacial defects account for the observed loss of strength [8,9,10]. Whereas the static strength decreases monotonically with rubber content, the influence of particle size is less regular: under dynamic loading, the peak strain decreases with increasing rubber content, with a maximum strength reduction of 52.3% reported for 30% rubber content and a 10 mm particle size, and the dynamic compressive strength of mixtures with particles larger than 5 mm is generally lower, as larger particles are associated with a more extensive ITZ [7].

While most experimental programmes on RuC have focused on rubber contents below 20–30% of the aggregate volume, fewer studies have explored the practical upper bound of rubber incorporation, at replacement levels of up to 50–60%. At such high contents, mechanical performance deteriorates markedly: a 60% replacement of fine aggregate by crumb rubber has been reported to reduce compressive strength, flexural strength, modulus of elasticity and density by approximately 32%, 36%, 43% and 21%, respectively, while nearly doubling the damping coefficient and increasing impact resistance by 75–170% [5,9,11]. Strength recovery strategies have been proposed to offset these losses: heat pre-treatment of the rubber combined with magnetised mixing water has recovered up to 74% of the lost compressive strength and increased impact resistance by up to 92% [12], while columns cast with 50% rubber content and externally strengthened with CFRP laminates have achieved load efficiencies exceeding those of unstrengthened conventional specimens [13,14,15]. Such high rubber contents therefore appear unsuited to unmodified load-bearing applications, but remain promising for lightweight, non-structural or damping-oriented uses, and can be rendered structurally viable through rubber pre-treatment or external strengthening.

In contrast to its detrimental effect on strength, rubber incorporation substantially improves ductility, toughness and impact resistance. Relative to conventional concrete, RuC exhibits superior energy absorption capacity at high loading rates, as the rubber particles absorb energy, retard crack propagation and delay progressive failure [7]. Three mechanisms have been identified: energy absorption through elastic deformation of the rubber particles, crack deflection through debonding at the rubber–matrix interface and stress relief associated with the induced micro-porosity [8]. The toughness index increases with rubber content, with no clear dependence on particle size, suggesting that energy absorption capacity is governed primarily by the amount of rubber, rather than its granulometry [7]. Post-test observations corroborate this enhanced ductility, with narrower, mesh-like crack patterns, no large fragment detachment and specimens that remain compact beyond peak stress, rather than failing abruptly [16].

A key observation emerging from the wider literature is that the detrimental effect of rubber on strength is considerably less pronounced at the structural level than at the material level [17]. Previous studies reported that increasing the crumb rubber content from 0% to 18% reduced the compressive strength of the mix by approximately 31%, whereas the ultimate capacities of beam and column specimens cast from the same concrete decreased by only about 6% and 12%, respectively. This disproportion between material- and member-level performance provides a strong incentive for investigating RuC directly in structural elements, rather than extrapolating member behaviour from small-scale material tests [18].

For flexural members, the effect of rubber has been examined through numerous four-point bending programmes on reinforced RuC beams. An extensive experimental programme involved testing full-scale self-consolidating RuC beams and found that increasing crumb rubber content enhanced beam curvature at service load, that replacement ratios of up to 20% improved curvature ductility and that higher rubber contents produced narrower, but more numerous, cracks [19]. Complementary studies have shown that, for a given compressive strength, reinforced beams cast with RuC exhibited ultimate flexural strength, cracking moment and load–deflection behaviour comparable to conventional beams, regardless of mix proportions and rubber content, indicating that existing code provisions for conventional concrete remain applicable, with equivalent accuracy, to predicting the cracking moment and ultimate flexural capacity of crumb rubber concrete beams [17,20].

The interaction between rubber content and longitudinal reinforcement has also been investigated. Tests on reinforced beams incorporating waste tyre rubber in fibre form at replacement ratios of 5%, 10% and 15% by volume of aggregate, combined with tension reinforcement of 8, 10 and 12 mm diameter, highlighted that increasing rubber content reduced the maximum load-carrying capacity, ultimate load, and energy absorption by up to approximately 62% at the highest replacement ratios, although moderate contents of up to 10% enhanced cracking resistance and deformability prior to fracture [20]. Similarly, experimental and numerical investigations on reinforced RuC beams with different longitudinal reinforcement ratios showed that an increase in reinforcement ratio enhanced load-carrying capacity, while reducing both midspan deflection and the ductility coefficient, for conventional and rubberised beams alike [17]. The substitution of natural aggregates by rubber produced only a slight decrease in load-carrying capacity, together with an increase in midspan deflection, a larger number of cracks prior to failure, and higher ductility coefficients for reinforcement ratios below 1.0%, confirming that RuC beams are more flexible than their conventional counterparts [17]. Strategies for mitigating the flexural penalties of rubber incorporation have also been proposed. Studies showed that, although the first visible flexural crack in a fully rubberised beam appeared at a lower load than in a plain concrete beam, the addition of steel fibres postponed crack initiation beyond the level of the conventional concrete beam and that the marginal reduction in initial flexural stiffness of RuC can be partly or fully recovered, either through steel fibre inclusion or through a functionally graded configuration, e.g., an upper layer of plain concrete over a lower layer of rubberised or steel fibre-reinforced RuC, which significantly improved the overall flexural response, both with and without fibres [21].

Taken together, the evidence spanning structural elements reveals a consistent pattern: moderate rubber replacement ratios, generally not exceeding 10–20%, preserve the load-bearing capacity of reinforced and composite members to a large extent while enhancing crack control, deformability and, under appropriate confinement or reinforcement configurations, ductility and energy dissipation. By contrast, the very high replacement levels of up to 50–60% discussed above remain, for the time being, largely confined to non-structural, lightweight or specifically strengthened applications. Systematic experimental data are nevertheless still scarce regarding the failure modes, load-carrying capacity and, in particular, the ductility of RuC members under the combined effect of rubber content and longitudinal reinforcement ratio. This knowledge gap currently hinders the confident large-scale application of waste tyre rubber in load-bearing structures.

In particular, the flexural behaviour of RuC beams incorporating high rubber contents has received comparatively little attention, and virtually no data is available on the interaction between high rubber replacement and high longitudinal reinforcement ratio configurations. Over-reinforced sections are conventionally associated with brittle, concrete-crushing failure preceded by limited warning, since the tension reinforcement remains elastic, while the compression zone governs collapse. The pronounced deformability and energy-dissipation capacity conferred by high rubber contents may, in principle, mitigate this brittleness, but the extent to which such beneficial interaction materialises at the section level has not been established. To address this gap, the present study investigates the flexural response of reinforced RuC beams cast with 40% and 60% replacement of small coarse aggregate (4–8 mm in diameter) by rubber particles, combined with longitudinal reinforcement ratios of 1.5% and 2.0%. This combination of parameters is intended to clarify whether, and to what extent, high rubber contents can offset the inherently brittle failure mode associated with highly reinforced sections, thereby providing experimental evidence to support the safer structural application of high-rubber-content RuC in flexural members.

## 2. Materials and Methods

### 2.1. Materials

A commercially available CEM I 42.5R cement was used, selected for its high early-age compressive strength. Rounded river gravel was used as the natural aggregate; the rounded particle shape reduces stress concentrations and delays the onset of early-age cracking [22,23].

Rubber aggregates were supplied by Radburg (Suceava, Romania), obtained by shredding end-of-life commercial vehicle tyres [24]. The resulting particles had a rougher surface texture than the natural aggregates. Before delivery, the rubber aggregates were sorted by maximum particle size and cleaned of impurities, including residual steel fragments and textile fibres from the shredding process. The rubber aggregates replaced 40% and 60%, by volume, of the small coarse aggregate fraction (4–8 mm) [25]. The 506 kg/m^3^ apparent density of rubber particles was experimentally determined and reported previously [5]. The rubber aggregates were not subjected to any pretreatment [26] before being used in the concrete mix.

Consistent with previous studies [5], the target compressive strength class for the concrete was C30/37 [27]. Based on preliminary laboratory testing, this strength class was identified as the most economical solution capable of achieving a compressive strength of at least 20 MPa for the rubberized concrete, at the standard curing age of 28 days, suitable for structural applications [28,29]. The concrete mix proportions, presented in Table 1, were supplied by a local ready-mix batching plant. For each concrete type, conventional concrete and rubberized concrete (RuC), a set of 15 cylindrical specimens (100 mm × 200 mm, diameter × height) was cast to evaluate the mechanical properties at 28 days, such as modulus of elasticity [30], compressive strength [31] and splitting tensile strength [32].

The longitudinal reinforcement used in the concrete beams was of type BST500C, with a yield strength f_y_ = 512 MPa and a modulus of elasticity E = 202 GPa [17].

### 2.2. Specimens

**Figure 1:**
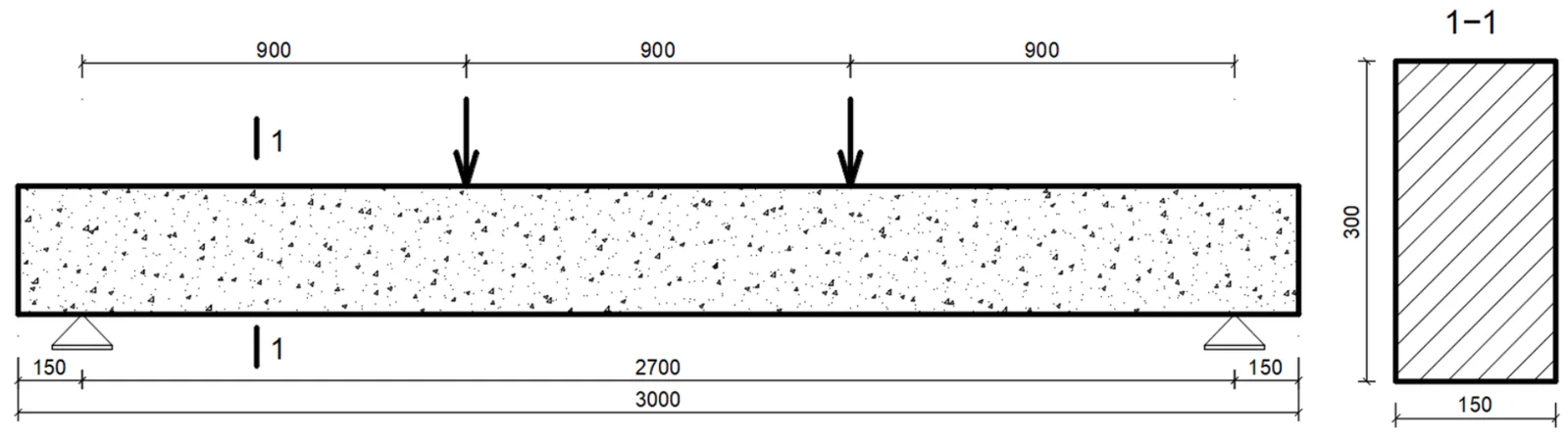
Geometrical dimensions of the reinforced concrete/rubberized concrete beams (dimensions in mm) [17].

**Figure 2:**
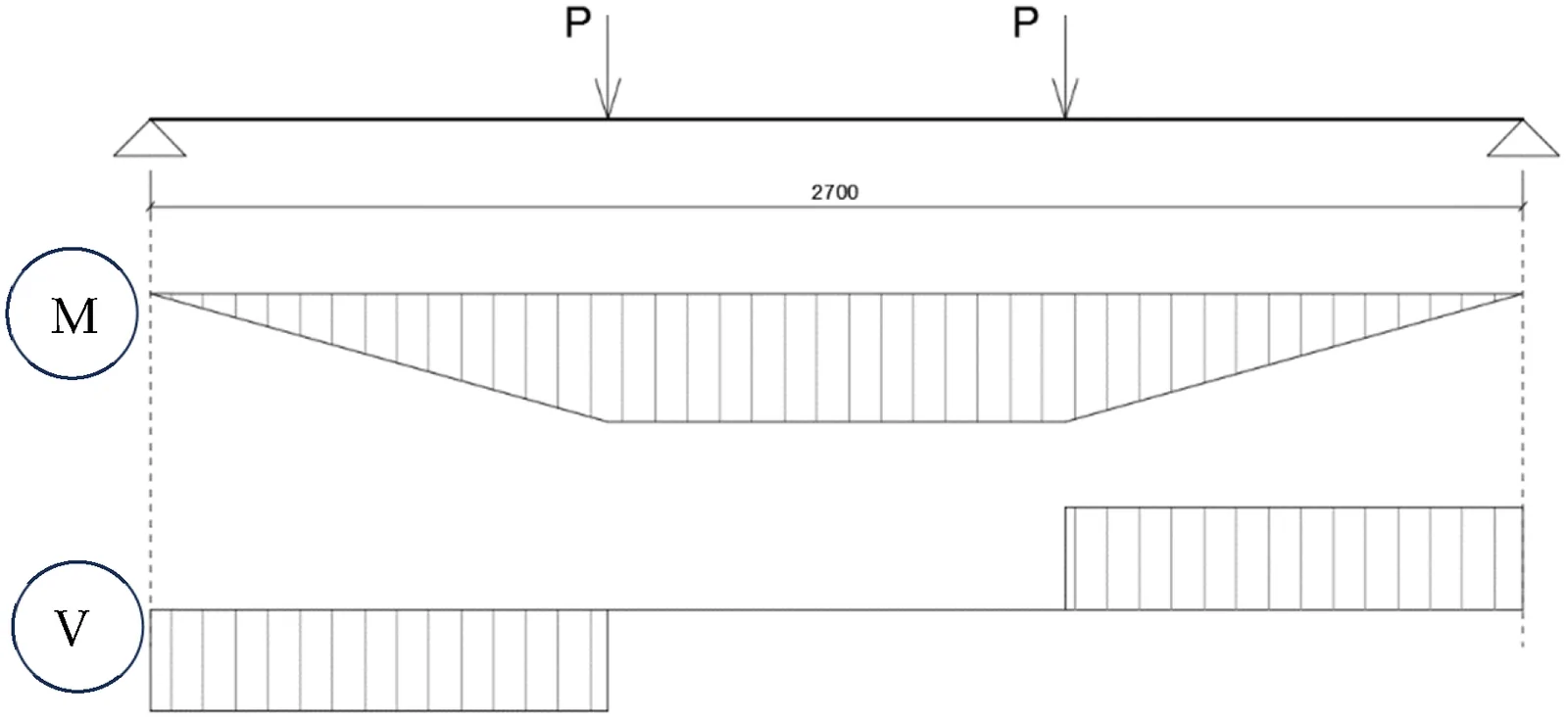
Loading diagram and diagrams of interval forces: bending moment (M); shear force (V).

**Figure 3:**
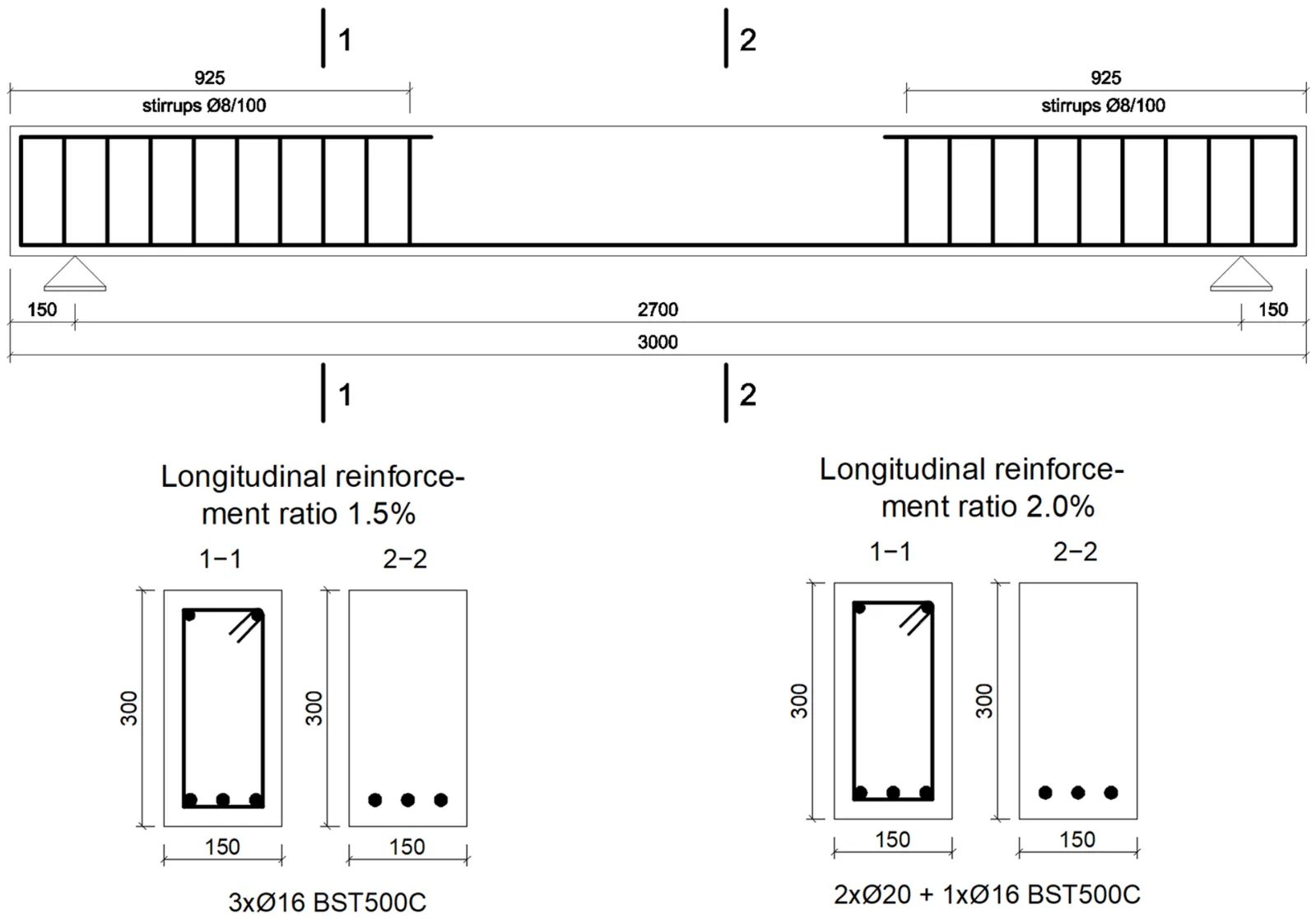
Reinforcement layout of the reinforced concrete/rubberized concrete beams (dimensions in mm).

The geometric dimensions of the beams considered in this study are presented in Figure 1. The target internal force diagrams are presented in Figure 2. The reinforcement layout, accounting for all longitudinal reinforcement ratios investigated (1.5% and 2.0%), is shown in Figure 3 [17]. Stirrups were omitted within the middle third of the clear span to isolate the flexural response of the beams, as shown in Figure 2. A comparable reinforcement configuration has previously been reported in the literature [33]. Within the two shear spans, stirrups were provided at a spacing of 100 mm, fabricated from OB37 low-carbon (mild) steel.

Reinforced concrete design codes conventionally mandate under-reinforced sections, ensuring tensile steel yielding precedes concrete crushing, thereby guaranteeing a ductile, forewarning failure mode. Highly reinforced sections (e.g., 1.5%, 2%) would instead fail via concrete crushing in compression while the reinforcement remains elastic, producing so-called compression-controlled members that typically exhibit brittle behaviour. Because such members are avoided in conventional design, owing to their sudden, low-warning failure, design codes generally discourage over-reinforced sections. Nevertheless, practical constraints, e.g., restricted section depth, can push detailing toward this regime. Prior studies have accordingly investigated methods of restoring ductility to over-reinforced members through compression zone modification [34].

Rubberized concrete (RuC) is independently recognised for enhancing precisely the toughness-related mechanisms that over-reinforcement compromises. The inclusion of tyre rubber has been shown to promote crack branching and stress redistribution during failure, restraining crack widening and yielding more ductile crack patterns compared to the relatively straight, brittle cracking observed in conventional concrete. Reinforced RuC beams incorporating waste tyre rubber have demonstrated measurable ductility improvements relative to control beams, with only modest reductions in ultimate flexural load [21,35].

**Figure 4:**
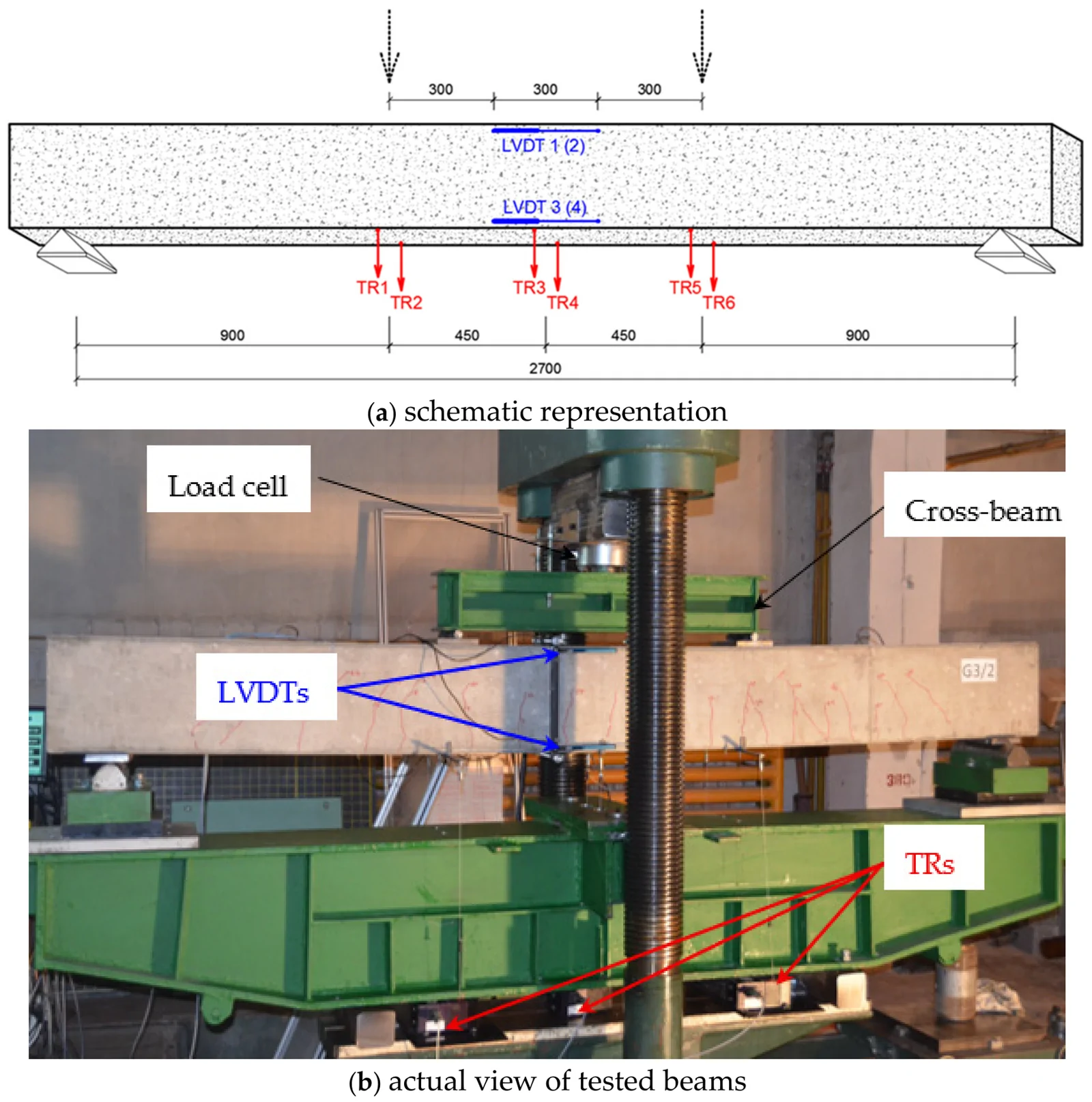
Experimental setup.

A 4-point loading test layout was selected to ensure that the midspan of the beams was subjected to pure bending. Similar layouts were also adopted in previous studies [36,37]. Linear variable displacement transducers (LVDTs), with a 0.001 mm accuracy, were mounted on both sides of the beams, spanning the middle third of the clear span (the constant-moment region) at the top and bottom fibres, to determine the average curvature within this region. Additional resistive-type displacement transducers (TR1–TR6), measuring 0.005 mm, were positioned on both sides of the beams at the midspan section and at the two load application points, as illustrated in Figure 4. Load was applied incrementally at a rate of 2 kN/min to allow real-time monitoring and marking of crack development corresponding to each load level. An ESAM Traveller CF data acquisition system was used to record all the data at a 10 Hz frequency. All beams had an effective depth of 265 mm and a shear span to effective depth ratio of 3.4.

Three beams were considered for each concrete mix shown in Table 1 and for each value of the longitudinal reinforcement ratio, for a total of 18 specimens. Of these 18 beams, the Ref and RuC40 specimens at the 1.5% reinforcement ratio (6 beams) were previously reported in [17], which examined Ref and RuC40 beams at 0.5%, 1.0% and 1.5% reinforcement. The remaining 12 beams, all RuC60 specimens at the 2.0% reinforcement ratio, constitute new experimental data not present in [17].

## 3. Results and Discussion

### 3.1. Material Properties

The experimentally determined values for the static modulus of elasticity in compression [30], compressive strength [31], splitting tensile strength [32] and peak axial strain are summarised in Table 2. The loading rate for the determination of static modulus of elasticity in compression and compressive strength was 4.5 kN/s, whereas, for splitting tensile strength, it was 1.8 kN/s. The value of the ultimate axial strain was assessed based on the full stress–strain curve of concrete in compression [38]. Out of the 15 cylinders cast for each mix, 10 were randomly selected for the determination of compressive strength, while the remaining five were used for splitting tensile strength.

The results in Table 2 confirm the well-documented reduction in stiffness and strength associated with rubber aggregate incorporation. Relative to the reference mix, the static modulus of elasticity decreased by approximately 9.5% and 12.3% for RuC40 and RuC60, respectively, while compressive and splitting tensile strengths declined proportionally, consistent with the lower stiffness and weaker interfacial bonding of rubber particles compared to natural aggregates [40,41].

This reduction is reflected in the corresponding concrete class downgrade from C45/55 (Ref) to C35/40 (RuC40 and RuC60), per Eurocode 2, Table 3.1 [39]. Notably, the experimentally determined ultimate axial strain (ε_cu,exp_) increased with rubber content, from 2.8‰ (Ref) to 3.4–3.5‰ (RuC40, RuC60), indicating enhanced deformability. This represents a consistent trend with energy-dissipating behaviour of rubber aggregates, previously reported [21,40]. These experimental values closely approximate the Eurocode 2 nominal ultimate strain limits, supporting the applicability of code-based deformation assumptions to rubberized concrete despite its modified compressive response.

### 3.2. Flexural Behaviour

#### 3.2.1. Force–Midspan Deflection Curves

**Figure 5:**
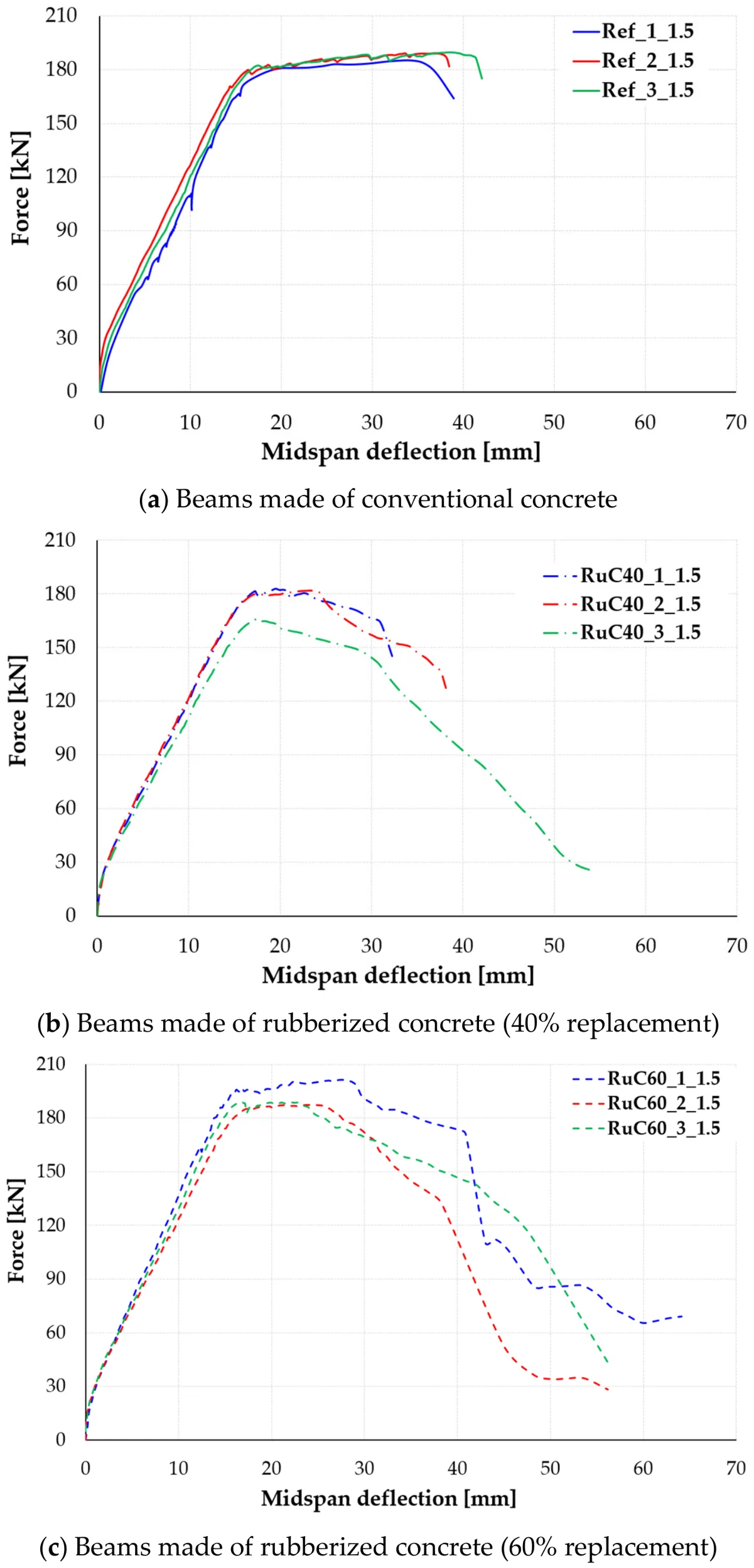
Load–midspan deflection curves for RC beams with 1.5% longitudinal reinforcement.

**Figure 6:**
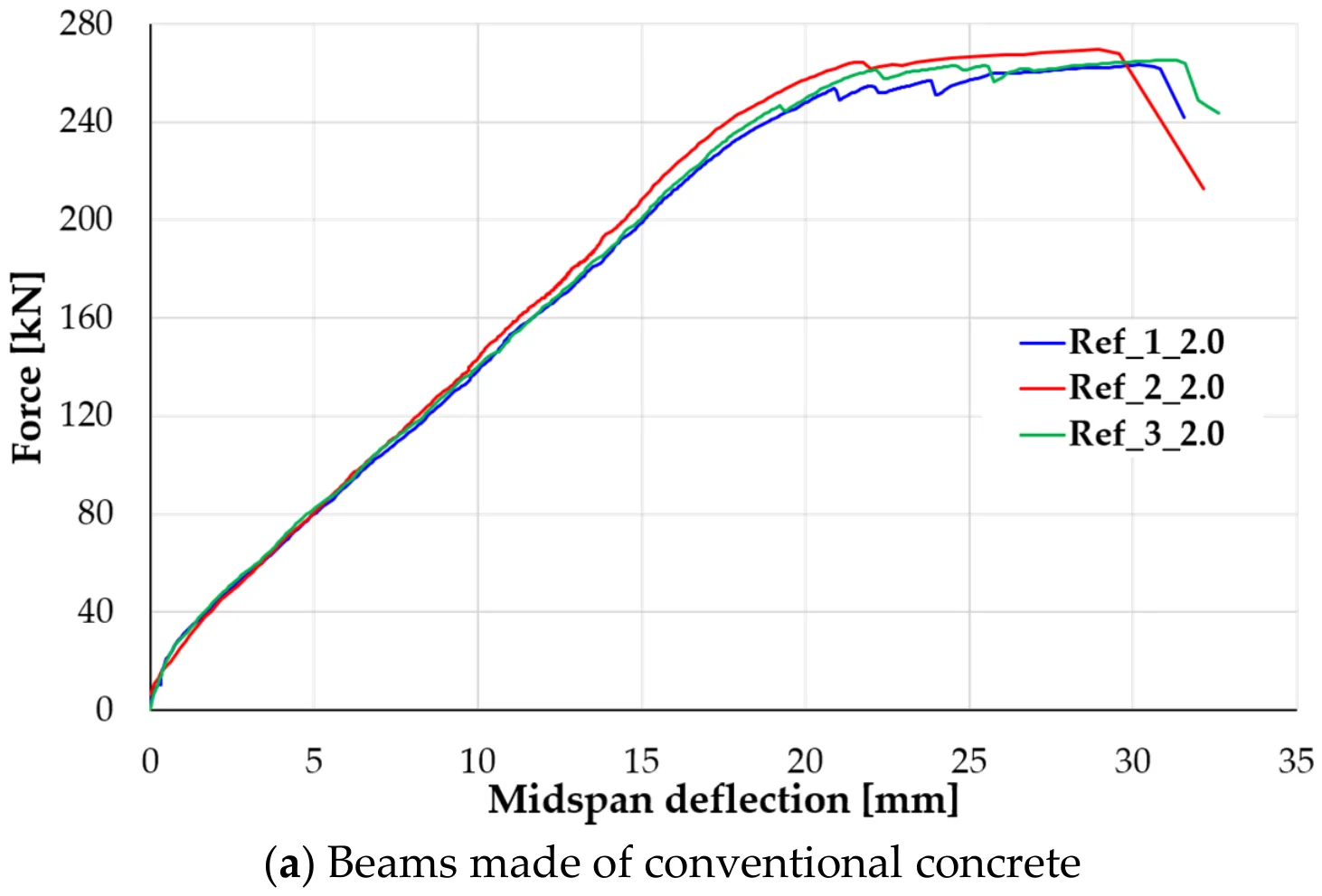

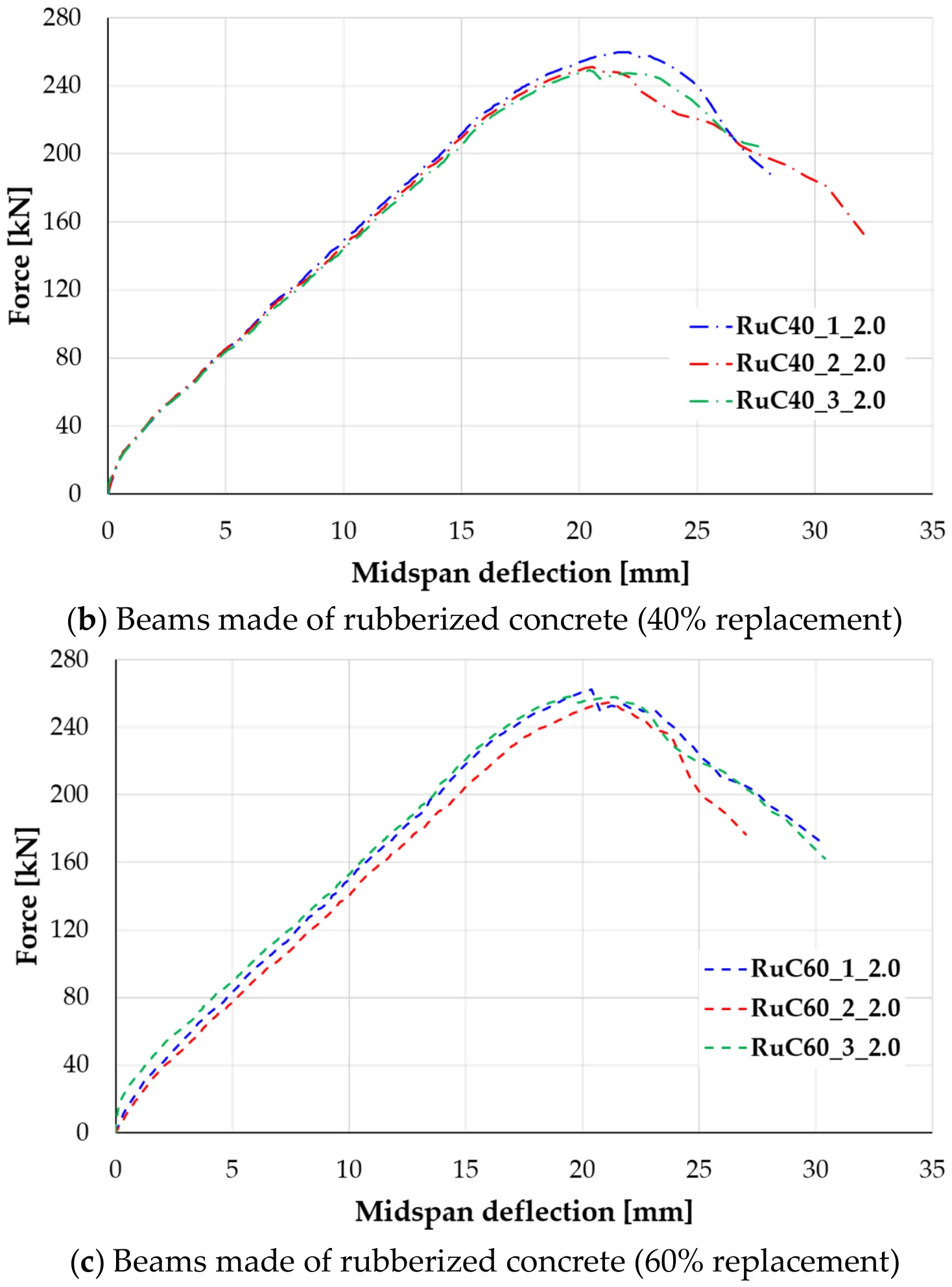
Load–midspan deflection curves for RC beams with 2.0% longitudinal reinforcement.

The force–midspan deflection curves of all specimens are presented in Figure 5, for a longitudinal reinforcement ratio of 1.5%, and in Figure 6, for a longitudinal reinforcement ratio of 2%.

The load–midspan deflection curves in Figure 5 reveal a clear trend of increasing deformation capacity with rubber content, alongside a corresponding reduction and greater dispersion in peak load. The conventional concrete beams (Ref) exhibited the stiffest, most consistent response among the three groups with limited post-peak ductility. Introducing 40% rubber replacement (RuC40) preserved a comparable peak load but produced greater scatter in post-peak behaviour. This is consistent with reports that reinforced rubberized concrete beams can exhibit measurable ductility gains relative to conventional beams, with only a modest accompanying reduction in ultimate flexural load [40]. At 60% replacement (RuC60), this trend became more pronounced than either previous scenario, with all three specimens exhibiting substantially extended post-peak deformation.

This progressive shift from a relatively brittle, plateau-type failure in conventional concrete toward a more gradual, energy-dissipating descent in the rubberized beams aligns with previous observations that rubber particle inclusion promotes crack branching and stress redistribution during failure, restraining crack widening and producing more ductile crack patterns than the comparatively straight, brittle cracking typical of conventional concrete [40]. Comparable toughness and displacement–ductility improvements have also been reported for functionally graded and fully rubberized beam configurations, further supporting the energy-absorbing role of dispersed rubber particles within the concrete matrix [21]. This behaviour is generally in line with the wider structural literature, which reports that flexural strength reductions in rubberized beams are typically smaller in magnitude than the corresponding reduction in compressive strength and that beams of similar compressive strength tend to exhibit similar flexural strength, cracking moment and load–deflection response, irrespective of rubber content or mix proportions [35,42], although increasing rubber content is consistently associated with greater inter-specimen variability in peak strength and post-peak response [42].

At the higher longitudinal reinforcement ratio (2.0%), as shown in Figure 6, the three concrete types exhibit markedly more comparable load–deflection responses than observed at 1.5%, consistent with the compression-controlled failure mode expected as sections approach the over-reinforced regime [43]. The conventional beams (Ref) reached the highest peak loads at deflections of 20–30 mm, sustaining a broad plateau before an abrupt strength loss beyond ~30 mm, characteristic of brittle concrete crushing once reinforcement ductility is no longer governing [44].

The RuC40 beams attained slightly lower peak loads at a comparable deflection (~20–22 mm) but displayed an earlier, more gradual post-peak descent extending to ~32 mm, indicating some retained toughness despite reduced peak capacity [40]. The RuC60 beams followed a similar peak load range and deflection capacity (~27–30 mm), with a comparable gradual softening trend. 

Unlike the 1.5% series, where increasing rubber content substantially extended deformation capacity, the 2.0% beams show convergent peak loads and deflection ranges across all three concrete types, suggesting that at higher reinforcement ratios, compression zone crushing, rather than rubber-induced crack bridging, increasingly governs failure, diminishing the relative influence of rubber content on post-peak ductility [35,43].

The values of the force corresponding to longitudinal reinforcement yielding (F_yield_), peak force (F_peak_) and corresponding midspan deflection (w) are summarised in Table 3.

Table 3 shows that yield and peak load capacity remained broadly comparable across the three concrete types at each reinforcement ratio, with differences generally within 5%, while deflection capacity at peak load (w_peak_) exhibited more pronounced, and less consistent, variation with rubber content.

At 1.5% reinforcement, w_peak_ increased progressively with rubber content, from 39.82 mm (Ref) to 41.50 mm (RuC40), and markedly to 58.93 mm (RuC60), a 48% increase relative to the reference beam. This indicates enhanced post-yield deformability consistent with the energy-dissipating role of rubber particles [21,45]. At 2.0% reinforcement, however, this trend reversed: w_peak_ decreased for both RuC40 (29.32 mm) and RuC60 (29.20 mm) relative to Ref (37.11 mm). This suggests that, at higher reinforcement ratios, compression zone crushing increasingly governs failure, limiting the influence of rubber-related toughening mechanisms observed at lower ratios [42]. 

This reinforcement ratio-dependent behaviour aligns with recent findings that rubberized concrete beam performance is sensitive to the interaction between tension reinforcement level and concrete-side deformation capacity [28], reinforcing the relevance of examining RuC beams across a range of reinforcement ratios, rather than relying solely on conventional, under-reinforced configurations.

#### 3.2.2. Cracking Patterns

**Figure 7:**
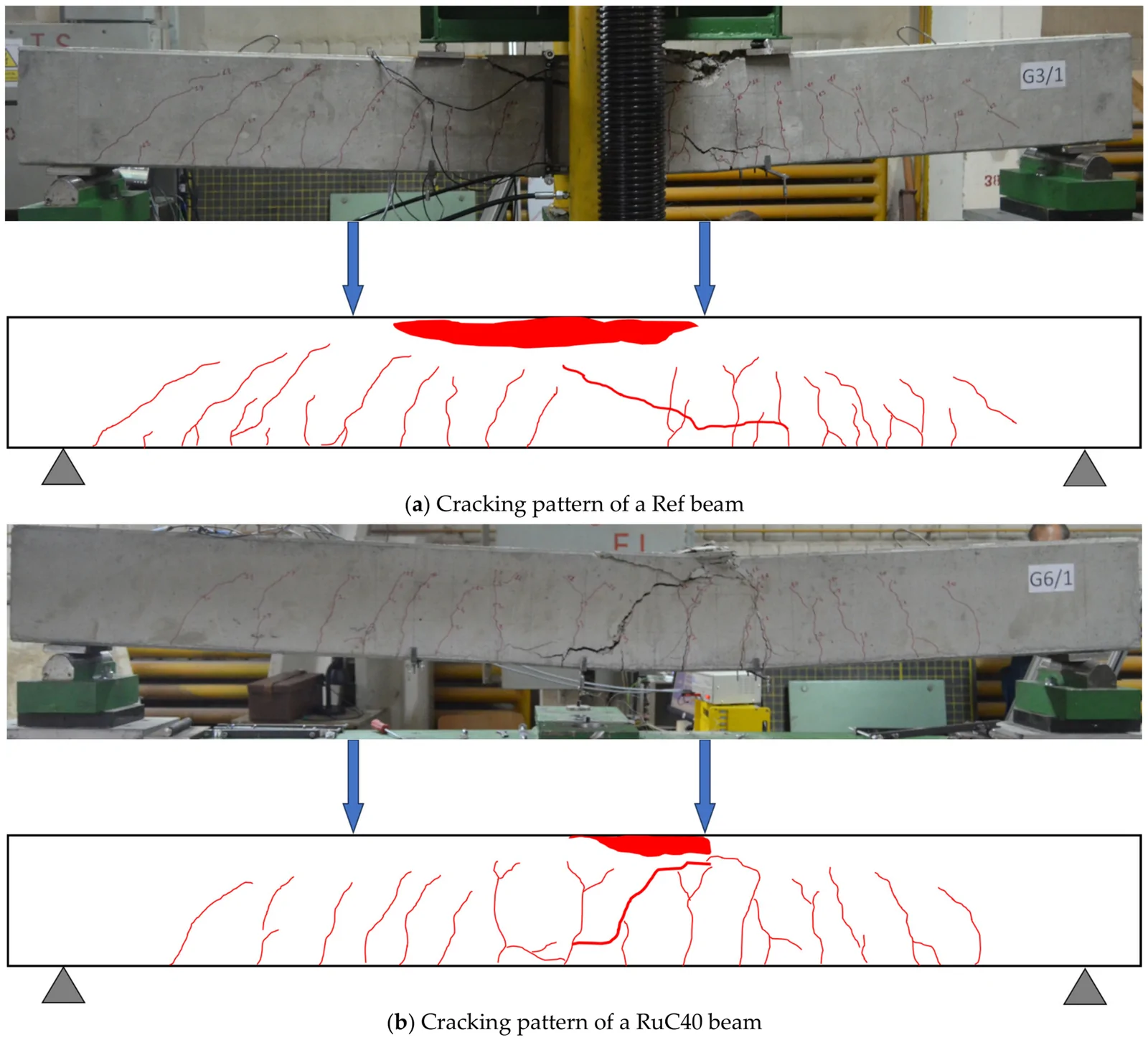

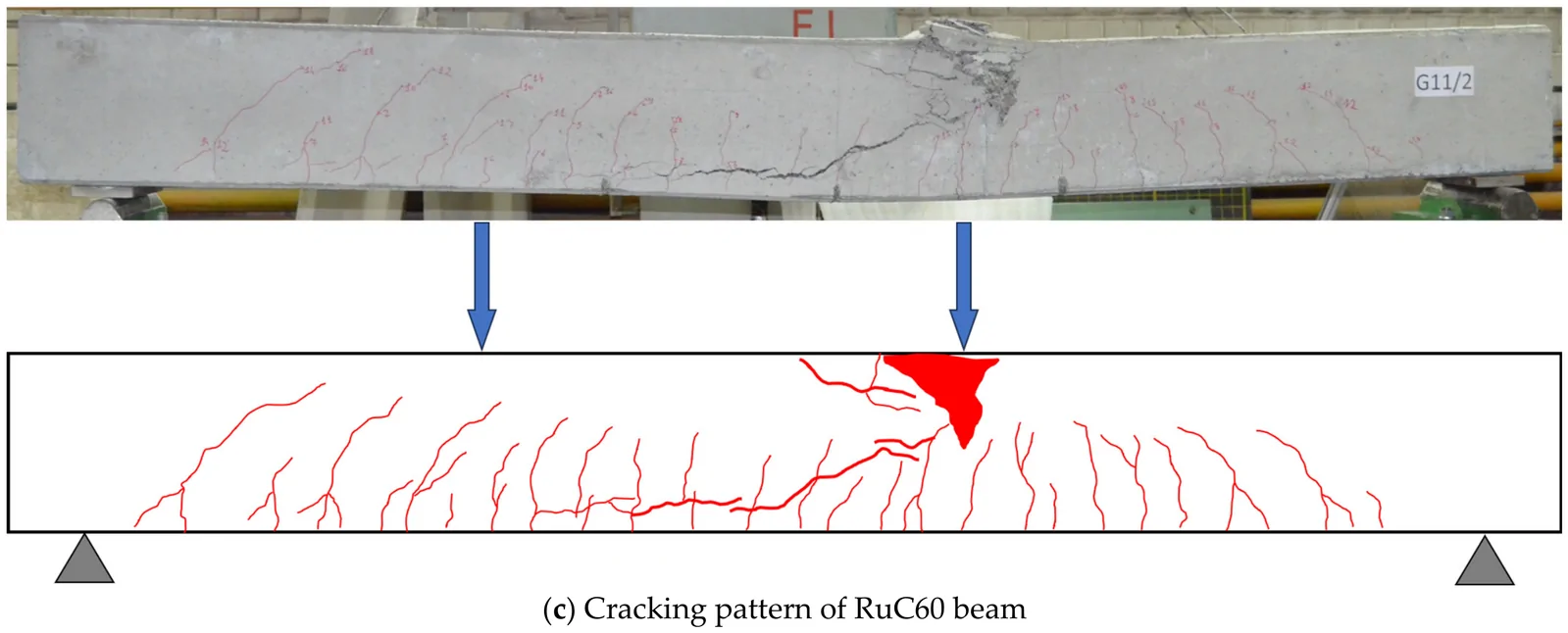
Crack patterns of beams with 1.5% longitudinal reinforcement ratio.

**Figure 8:**
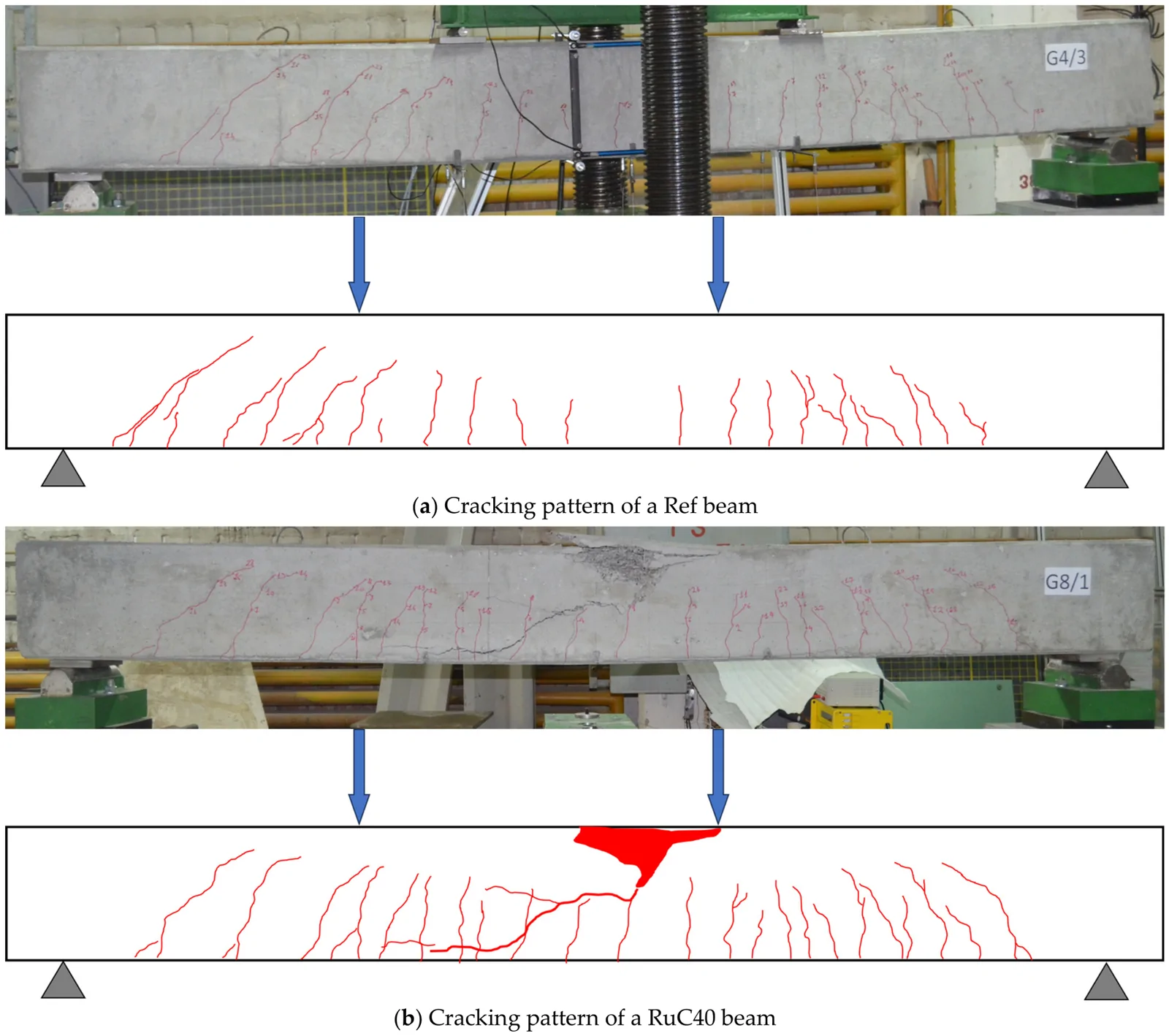

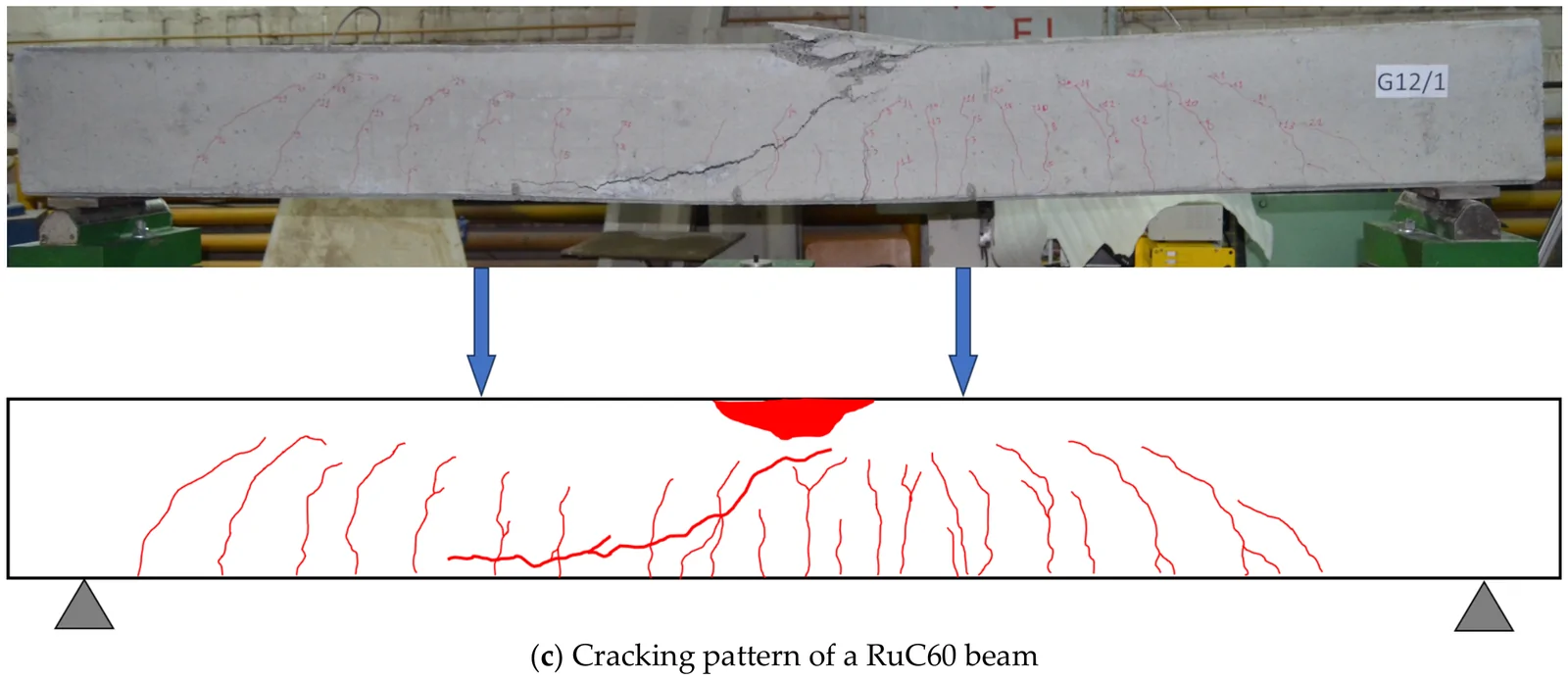
Cracking patterns of beams with 2.0% longitudinal reinforcement ratio.

The cracking patterns of each considered scenario are presented in Figure 7, for a longitudinal reinforcement ratio of 1.5%, and in Figure 8, for a longitudinal reinforcement ratio of 2%.

The crack patterns presented in Figure 7 and Figure 8 exhibit the expected flexural cracking distribution within the shear spans, with inclined cracks propagating upward from the tension face and converging toward the top-fibre compression zone at midspan, consistent with the stirrup-free configuration adopted to isolate flexural behaviour. The red-shaded areas denote regions of crushed concrete, marking the extent and location of the compression failure zone, while the thick horizontal lines running along the level of the longitudinal reinforcement represent debonding cracks, indicating a local loss of bond between the tension steel and the surrounding concrete once the compression zone approaches failure. Such bond deterioration along the reinforcement axis is known to significantly affect the strength, stress–strain state, and failure mode of reinforced concrete beams in bending, independent of rubber content [46].

At the 1.5% reinforcement ratio (Figure 7), all three concrete types display a dense, well-distributed flexural crack pattern in the shear spans; however, the extent of the crushed concrete zone and the presence of horizontal debonding cracks vary with rubber content. The RuC40 and RuC60 beams exhibit a more pronounced horizontal debonding crack extending from the crushed region toward one of the load points, suggesting that increased rubber content is associated with more localised bond deterioration once the compression zone begins to fail. This is consistent with previous reports that concrete strain distribution, neutral axis depth, and cracking behaviour are measurably altered by rubber content in reinforced rubberized concrete beams [42].

At the 2.0% ratio (Figure 8), the reference beam shows a comparatively sparse crack distribution near midspan, without a pronounced localised crushing zone or debonding crack, whereas both RuC40 and RuC60 beams develop a clearly defined, funnel-shaped crushed concrete region accompanied by a distinct horizontal debonding crack extending several hundred millimetres along the reinforcement level. This is consistent with the compression-dominated failure mechanism expected as sections approach the over-reinforced range and bond demand along the tension steel intensifies near failure [42,46].

#### 3.2.3. Initial Stiffness and Ductility

The initial (pre-cracking) stiffness, computed as the secant slope of the force–deflection curve between 5% and 30% of the peak load, proved largely insensitive to rubber content, averaging 17.3–19.5 kN/mm at 1.5% reinforcement and 17.7–19.2 kN/mm at 2.0% reinforcement across Ref, RuC40 and RuC60. Although rubber substitution reduced the static modulus of elasticity of concrete by up to 12% [10,41], this had only a marginal effect on the composite flexural stiffness, since the reinforcement increasingly governs the uncracked elastic response of the section. This behaviour is consistent with observations reported for other reinforced rubberised concrete beams [35].

Because the three mixes reach markedly different peak loads and deflection capacities, comparing the raw force–deflection slope conflates the (small) differences in composite flexural rigidity with the much larger role that each beam’s own strength and deformability play in setting the practical range over which that rigidity is mobilised. To isolate the shape of the initial response, independent of absolute capacity, the branch was reconsidered in dimensionless terms, F/F_peak_ versus w/w_peak_ (where w denotes the midspan deflection). The same rationale underlying the displacement ductility index is routinely used in reinforced concrete assessment, where deformation is normalised against a specimen-specific reference, rather than compared in absolute units [47]. On this basis, the normalised angle, θ_norm_ = arctan[(F/F_peak_)/(w/w_peak_)], decreased consistently from Ref (63.5°) to RuC40 (57.9°) to RuC60 (55.6°) and at 1.5% and 2.0% reinforcement, respectively. This indicates that rubberised beams mobilise proportionally more of their eventual deflection capacity earlier in loading than Ref beams, consistent with previous reports that increasing rubber content reduces flexural rigidity relative to ultimate deformability [35,41]. Unlike the conventional displacement ductility coefficient, which contrasts deflection at peak load against deflection at yield to characterise post-yield deformation capacity [47], θ_norm_ characterises the shape of the pre-yield branch itself, offering a stiffness-type measure that remains comparable across specimens of differing absolute capacity.

Ductility denotes the capacity of a member to undergo inelastic deformation without a significant reduction in load-carrying capacity prior to failure. This parameter is fundamental to quantifying the energy absorption capacity of beams and to assessing the available structural safety margin [48]. A commonly adopted approach for assessing beam ductility is the displacement ductility index, defined as the ratio of the midspan deflection at ultimate load to the midspan deflection at the onset of yielding. The corresponding displacement ductility coefficients are summarised in Table 4.

The ductility coefficients in Table 4 reflect the deflection trends reported in Table 3. At 1.5% reinforcement, RuC60 exhibited the highest ductility (2.52), consistent with its markedly larger peak load deflection (58.93 mm) relative to a comparable yield deflection, while RuC40 showed the lowest value (2.26) despite a peak load close to the reference beam, reflecting its more limited post-yield deformation gain. At 2.0% reinforcement, ductility values converged across all three concrete types (1.40–1.47), mirroring the comparable w_yield_ and w_peak_ values shown in Table 3 and confirming that higher reinforcement ratios suppress the deformation capacity benefits observed at 1.5%, regardless of rubber content, as compression zone crushing increasingly governs failure.

The non-monotonic variation in displacement ductility stems from the rates of increase between yield and peak deflections: at 40% replacement, reduced section stiffness results in a 17% increase in the corresponding deflection (with respect to Ref case, Table 3) which is more than the modest gain in peak deflection of only 4%. On the other hand, a substantial 48% increase in peak deflection was observed at 60% replacement, compensating for its higher yield deflection increase (43% with respect to Ref case, Table 3), thereby restoring and enhancing overall ductility.

## 4. Conclusions

This study experimentally investigated the flexural behaviour of reinforced rubberized concrete (RuC) beams, incorporating high rubber contents (40% and 60% replacement of the small coarse aggregate fraction by volume) and two longitudinal reinforcement ratios (1.5% and 2.0%), approaching the over-reinforced regime. Based on the material characterisation and structural testing presented, the following conclusions can be drawn:

1. Rubber aggregate incorporation reduces the static modulus of elasticity, compressive strength, and splitting tensile strength relative to conventional concrete, corresponding to a Eurocode 2 concrete class downgrade from C45/55 (Ref) to C35/40 (RuC40 and RuC60). In turn, it increases the peak axial strain, indicating enhanced deformability of the rubberized mixes prior to peak compressive stress.
2. At the lower reinforcement ratio (1.5%), increasing rubber content progressively enhances deformation capacity at peak load, with RuC60 beams exhibiting a midspan deflection approximately 48% greater than the reference beams, while yield and peak load capacities remained broadly comparable (within 5%) across all mixes.
3. At the higher reinforcement ratio (2.0%), the rubber-related toughening effect is suppressed: deflection capacity at peak load decreases for both RuC40 and RuC60 relative to the reference beams, indicating that compression zone crushing increasingly governs failure as sections approach the over-reinforced range.
4. Displacement ductility coefficients corroborate these deflection trends, with RuC60 beams achieving the highest ductility (2.52) at 1.5% reinforcement. All three concrete types converge to comparable, markedly lower ductility values (1.40–1.47) at 2.0% reinforcement.
5. Crack pattern observations, including the extent of concrete crushing and horizontal debonding cracks along the reinforcement axis, are consistent with the load–deflection and ductility findings, confirming a shift toward compression-dominated, bond-sensitive failure at the higher reinforcement ratio.

Overall, the results indicate that the structural benefits of high rubber content in RuC beams, namely enhanced deformation capacity and ductility, are most pronounced in under-reinforced configurations and diminish as the longitudinal reinforcement ratio increases toward the over-reinforced range. Here, concrete compressive behaviour becomes the dominant factor governing failure. These findings support the potential structural use of high-rubber-content RuC in flexural members with moderate reinforcement ratios, while highlighting the need for caution, or supplementary confinement/strengthening measures, when such mixes are used in more heavily reinforced sections. Future work should extend this investigation to additional reinforcement ratios, long-term durability performance and validation through numerical modelling of the observed bond slip and compression zone failure mechanisms supported by X-ray computed tomography investigations [49]. However, since such tests will create a costly multidimensional test space, the authors will also investigate alternative solutions for prioritising informative evaluations [50].

## Figures and Tables

**Table 1:** Mix proportions of conventional (Ref) and rubberized concrete (RuC40, RuC60).

Concrete	Cement	Water	Superplasticizer	Sand	Coarse Aggregates		
					4−8 mm	4−8 mm Rubber	8−16 mm
	[kg/m^3^]	[l/m^3^]	[l/m^3^]	[kg/m^3^]	[kg/m^3^]	[kg/m^3^]	[kg/m^3^]
Ref	489	198.3	2.4	582	388	−	646.7
RuC40					233	43.4	
RuC60					155.2	65.05	

**Table 2:** Material characteristics.

Material	E_c_ *	f_c_′ **	f_c,t_ ***	ε_cu,exp_ **	ε_cu_ [39]	Concrete Class [39]
	[GPa]	[MPa]	[MPa]	[‰]	[‰]	
Ref	37.81	57.37	4.00	2.8	3.5	C45/55
RuC40	34.22	46.37	3.47	3.4	3.5	C35/40
RuC60	33.17	44.82	3.37	3.5	3.5	C35/40

* average of 15 determinations. ** average of 10 determinations. *** average of 5 determinations.

**Table 3:** Performance parameters of RC beams.

Specimen	F_yield_			F_peak_			w_yield_			w_peak_		
	Value	StDev	COV	Value	StDev	COV	Value	StDev	COV	Value	StDev	COV
	[kN]	[kN]	[%]	[kN]	[kN]	[%]	[mm]	[mm]	[%]	[mm]	[mm]	[%]
Longitudinal reinforcement ratio of 1.5%												
Ref [17]	180.43	2.01	1.11	187.99	2.44	1.30	16.39	1.12	6.83	39.82	1.96	4.93
RuC40 [17]	172.83	7.44	4.30	176.96	9.47	5.35	18.38	1.67	9.09	41.51	3.31	7.98
RuC60	186.60	1.17	0.63	188.13	1.08	0.57	23.38	2.09	8.94	58.93	4.52	7.66
Longitudinal reinforcement ratio of 2.0%												
Ref	259.90	3.99	1.54	265.99	3.19	1.20	25.24	0.47	1.86	37.11	0.54	1.68
RuC40	251.21	4.87	1.94	253.29	5.74	2.27	20.94	1.65	7.88	29.32	2.35	8.01
RuC60	253.67	4.02	1.58	258.32	3.79	1.47	20.56	1.33	6.47	29.20	1.90	6.51

**Table 4:** Ductility of RC beams (average values ± standard deviation).

Longitudinal Reinforcement Ratio	Ref	RuC40	RuC60
1.5%	2.43 ± 0.04	2.26 ± 0.12	2.52 ± 0.05
2.0%	1.47 ± 0.04	1.40 ± 0.10	1.42 ± 0.04

## Data Availability

Data is available from the corresponding authors based on reasonable requests.
